# *De novo* sequencing and analysis of root transcriptome using 454 pyrosequencing to discover putative genes associated with drought tolerance in *Ammopiptanthus mongolicus*

**DOI:** 10.1186/1471-2164-13-266

**Published:** 2012-06-21

**Authors:** Yijun Zhou, Fei Gao, Ran Liu, Jinchao Feng, Hongjie Li

**Affiliations:** 1 College of Life and Environmental Sciences, Minzu University of China, Beijing, 100081, China; 2 Biotechnology Research Institute, Chinese Academy of Agricultural Sciences, Beijing, 100081, China; 3 The National Key Facility for Crop Gene Resources and Genetic Improvement (NFCRI), Institute of Crop Sciences, Chinese Academy of Agricultural Sciences, Beijing, 100081, China

**Keywords:** *Ammopiptanthus mongolicus*, Drought, Root, Transcriptome, 454 pyrosequencing

## Abstract

**Background:**

*De novo* assembly of transcript sequences produced by next-generation sequencing technologies offers a rapid approach to obtain expressed gene sequences for non-model organisms. *Ammopiptanthus mongolicus*, a super-xerophytic broadleaf evergreen wood, is an ecologically important foundation species in desert ecosystems and exhibits substantial drought tolerance in Mid-Asia desert. Root plays an important role in water absorption of plant. There are insufficient transcriptomic and genomic data in public databases for understanding of the molecular mechanism underlying the drought tolerance of *A. mongolicus*. Thus, high throughput transcriptome sequencing from *A. mongolicus* root is helpful to generate a large amount of transcript sequences for gene discovery and molecular marker development.

**Results:**

A total of 672,002 sequencing reads were obtained from a 454 GS XLR70 Titanium pyrosequencer with a mean length of 279 bp. These reads were assembled into 29,056 unique sequences including 15,173 contigs and 13,883 singlets. In our assembled sequences, 1,827 potential simple sequence repeats (SSR) molecular markers were discovered. Based on sequence similarity with known plant proteins, the assembled sequences represent approximately 9,771 proteins in PlantGDB. Based on the Gene ontology (GO) analysis, hundreds of drought stress-related genes were found. We further analyzed the gene expression profiles of 27 putative genes involved in drought tolerance using quantitative real-time PCR (qRT-PCR) assay.

**Conclusions:**

Our sequence collection represents a major transcriptomic resource for *A. mongolicus*, and the large number of genetic markers predicted should contribute to future research in *Ammopiptanthus* genus. The potential drought stress related transcripts identified in this study provide a good start for further investigation into the drought adaptation in *Ammopiptanthus.*

## Background

Desert ecosystems currently cover at least 35% of the Earth’s land surface and, in China, the area of desert land amounts to approximately 2,080 million km^2^, covering 22% of total land area of the country [1]. Furthermore, the desert region worldwide is still expanding partly due to the ongoing global warming. Conservation of the genetic resources of endemic desert plants is critical to global efforts to curb desertification, prevent further deterioration of the fragile ecosystems in arid and semi-arid regions, and maintain biodiversity in deserts. *Ammopiptanthus*, the only genus with evergreen broadleaf habit in the desert and arid regions of Mid-Asia, including Northern China, plays a critical role in maintaining desert ecosystems and delaying further desertification. A deeper understanding of the genetic control of adaptation to desert environment in *Ammopiptanthus* would be beneficial and timely.

According to fossil evidence, the vegetation in northwestern China was dominated by evergreen broadleaf forest in the early Tertiary period, but with the climate becoming drier and colder in central Asia, the forest was gradually replaced by steppe and then by desert [2]. *Ammopiptanthus* is a relict survivor of the evergreen broadleaf forest of this region from the Tertiary period possibly owing to its high tolerance to drought and cold.

The genus *Ammopiptanthus* (Leguminosae) comprises of two species: *Ammopiptanthus mongolicus* (Maxim. ex Kom.) Cheng f. and *Ammopiptanthus nanus* (M. Pop.) Cheng f. In China, *A. mongolicus* mainly distributes in the desert and arid regions of Inner Mongolia and Ningxia Autonomous Regions, as well as Gansu Province. *A. mongolicus* is one of the constructive species of desert ecosystems and serves a vital function in maintaining desert vegetation. The habitats of *A. mongolicus* are stony and/or sandy deserts with an annual precipitation ranging from 100 mm to 160 mm and a mean annual potential evaporation up to 3,000 mm. To adapt to the harsh environment, *A. mongolicus* have developed sophisticated mechanisms to maintain the capacity of water absorption from soil. The deep flourishing root system is essential in the high drought tolerance of *A. mongolicus*; however, the genetic mechanism is still unknown. Because of the ecological importance and the high academic value in *A. mongolicus*, several studies have focused on anatomy and physiology [3], genetic marker and diversity [1,4], freeze resistance protein [5] and cold tolerance mechanisms [6], and transgenic functional analysis of *AmNHX2*[7], *AmLEA*[8], and *AmCBL1*[9]. Few studies have addressed the drought tolerance mechanism of *A. mongolicus* except that Xu *et al.* reported that more osmolyte was found in drought-stressed *Ammopiptanthus* leaves [10].

A large number of nucleotide sequences are prerequisite for identifying drought related genes and further understanding the molecular mechanism underlying drought tolerance of *A. mongolicus*. However, little resources exist for *A. mongolicus* in GenBank (749 EST and 125 Nucleotide sequences prior to 1 Sept. 2011) and *A. nanus*, another species in the genus *Ammopiptanthus*, despite of the importance of the genus. Considering the large genome size of the woody plants, whole genome sequencing of *A. mongolicus* is difficult. The construction of large EST collections is thus the most promising approach for providing functional genomic level information in *A. mongolicus*. Sequencing and analysis of ESTs is one of primary tools for discovery of novel genes, especially in non-model plants. In addition, ESTs can also be used for other functional genomic projects, including gene expression profiling, microarrays, molecular markers development, and physical mapping [11,12].

In recent years, next-generation sequencing (NGS) technologies, including Roche/454 pyrosequencing, Illumina/Solexa sequencing technology, and Applied Biosystems SOLiD sequencing technology, have led to a revolution in genomics and provided cheaper and faster delivery of sequencing information [13]. The newest 454 sequencing platform, the GS FLX Titanium, can generate one million reads with an average length of up to 400 base pairs (bp) at 99.5% accuracy per run. The 454 sequencing platform has been successfully applied in transcriptome sequencing of *Brassica napu*s [14], *Artemisia annua*[15], *Eucalyptus grandis*[16], *Olea europaea*[17], *Arabidopsis thaliana*[18,19], *Medicago truncatula*[20], and other plant species [21]. To date, the 454 pyrosequencing technique is the most widely used NGS technology for the *de novo* sequencing and analysis of transcriptomes in non-model organisms.

Simple sequence repeat (SSR) markers are microsatellite loci that can be amplified by polymerase chain reaction (PCR) using primers designed for unique flanking sequences. Compared with other types of molecular markers, SSRs have many advantages, such as simplicity, effectiveness, abundance, hypervariability, reproducibility, codominant inheritance, and extensive genomic coverage [22]. Based on the original sequences used to identify the simple repeats, SSRs can be divided into genomic SSRs and EST-SSRs. Genomic SSR markers have some disadvantages. Firstly, genomic SSR markers are derived from genomic BAC library, most of which come from the intergenic regions with no gene function. Secondly, the procedures for developing such markers are difficult, complex, and high-cost. In addition, the interspecific transferability of genomic SSRs is limited because of either a disappearance of the repeat region or degeneration of the primer binding sites [23]. Alternatively, EST-SSRs are derived from expressed sequences, which are more evolutionary conserved than noncoding sequences; therefore, EST-SSR markers have a relatively high transferability [24]. With the increasing number of ESTs deposited in public databases, an expanding number of EST-SSRs have been developed, and the polymorphism and transferability of EST-SSRs have been evaluated in many plant species [24-29]; however, there is no report on development of EST-SSR markers in *A. mongolicus* by now*.*

In order to significantly expand the transcript catalog of *A. mongolicus*, identify candidate genes involved in drought tolerance, and develop more SSR markers, we performed large-scale transcriptome sequencing of *A. mongolicus* root using Roche/454 next-generation sequencing technology. A total of 672,002 root-specific ESTs were obtained and assembled into 29,056 unique sequences. Bioinformatics analysis indicated that these unique sequences represent at least 9,771 protein coding transcripts. Thousands of potential simple sequence repeats molecular markers are discovered and 27 genes that were differentially expressed under drought treatment were identified by further quantitative real-time PCR analysis. This study will provide novel insights into the molecular mechanism underlying the drought tolerance in *A. mongolicus*.

## Results

### 454 sequencing of the *Ammopiptanthus* root transcriptome

**Figure 1:**
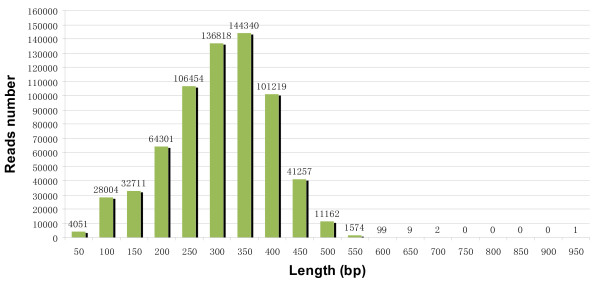
Size distribution of the 454 HQ reads.

A cDNA library constructed by SMART technology from the pooled RNA from drought-stressed and unstressed root samples of *A. mongolicus* was subjected to a half plate run with the 454 GS FLX Titanium platform. This half plate run produced 672,002 high quantity (HQ) reads with an average sequence length of 279 bp (SD = 92.2, range = 40–902) (Table 1). Of the HQ reads, 81.8% were over 200 bp in length, and 44.6% were over 300 bp in length. The size distribution of the reads is shown in Figure 1. All HQ reads were also deposited in the National Center for Biotechnology Information (NCBI) and can be accessed in the Short Read Archive (SRA) under the accession number SRX142053.

**Table 1:** Overview of the sequencing reads and reads after preprocessing

Statistics	
Sequencing reads before preprocessing	
Number of high-quality (HQ) reads	672,002
Average length of HQ read (bp)	279 ± 92.2
Total length (bp)	187,354,158
Reads after trimming and preprocessing	
Number of reads used for assembly	654,834
Average read length (bp)	272 ± 88.5
Total length (bp)	178,088,655

**Figure 2:**
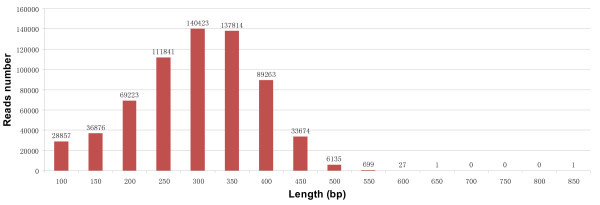
Size distribution of the sequencing reads used for assembly.

Prior to assembly, the low quality reads, adapter/primer sequences and sequences of less than 50 bp were removed using SeqClean (latest version) and Lucy (1.20p) first, and then Newbler v2.5.3. As a result, a total of 654,834 (97.4% of total HQ reads) sequencing reads was used for *de novo* assembly. The length distribution of these sequencing reads is shown in Figure 2.

### *De novo* assembly of sequencing data using three assemblers and comparison of the assemblies

To get a better assembly result, three assembly programs, Newbler (version 2.5.3), Mira (version 3.2.1) and Cap3 with default or optimized parameters were used for *de novo* assembly of our 454 sequencing data. We aimed at more long contigs and more contigs with homologs in soybean protein database (Phytozome v7.0, http://www.phytozome.net/).

We first run assemblies using the three assemblers with their default parameters, and similar assembly results were obtained in assemblies using Mira and Cap3. However, remarkably less contigs quantity and less contigs with homologs in soybean protein database were shown in the assemblies using Newbler with default parameters (Table 2). To increase the number of reads used in the assembly and get more amount of contigs, we then run assemblies using Newbler with a set of optimized parameters according to the assembler manual by checking “Use duplicate reads”, “Extend low depth overlaps”, “Reads limited to one contig”, and “Single Ace file” options.

**Table 2:** Assembly results of 454 data using various programs with default or optimized parameter

Parameter	Cap3	Newbler	Newbler (optimized parameter)	MIRA
Number of contigs	16,114	5,886	15,173	20,744
Total bases of contigs (bp)	7,665,534	4,380,563	7,348,496	9,060,888
Number of Contig ≥ 1000 bp	1,028	1,286	1,135	1,138
Contig N50 (bp)	462	948	491	436
Mean contig length (bp)	475.71	744.23	484.31	436.80
Number of singlets	19,605	72,556	13,883	67,537
Total bases of singlets (bp)	4,587,902	18,194,876	2,609,778	16,693,077
Total number of reads used in the assembly	635,229	582,278	640,951	587,297
	94.68%	86.65%	95.38%	87.40%
Contigs with significant hits^a^	8,414	3,696	5,238	11,003
	52.22%	62.79%	34.52%	53.04%
Contigs with 80% or greater coverage^b^	58	65	84	74
	0.3599%	1.104%	0.5536%	0.3567%
Soybean protein hits^c^	5,332	2,596	3,657	5,528
	9.558%	4.653%	6.560%	9.909%
Soybean proteins with 80% or greater coverage^d^	44	60	84	66
	0.0079%	0.1076%	0.1506%	0.1183%

^a^ Contigs showing significant hits (E < 1e-5) with soybean proteins. ^b^ Contigs showing 80% or greater coverage of soybean proteins. ^c^ Unique soybean proteins to which contigs show significant hits (E < 1e-5). ^d^ Unique soybean proteins to which contigs show 80% or greater coverage.

We compared the four assemblies using the following standard metrics: total number of reads used in the assembly, number of contigs generated, N50 length of contigs, number of contigs, mean contig length, and summed contig length. We also evaluated assembly integrity and completeness by comparing with the soybean protein datasets (Table 2).

Ideally, the optimal assembler will use almost all the reads given. In this respect, Newbler (optimized parameter) behaved best, and then Cap3 and Newbler, and MIRA use the least reads. The optimal assembler will produce the longest summed length of contigs, with a relatively longer mean contig length, while avoiding over-assembly of reads into *in silico* chimaeras. Although Newbler with default parameter generated an assembly with the largest N50, mean contig length and number of contig no less than 1,000 bp, it also produced the smallest summed length of contigs, and startlingly low total number of contigs. MIRA with default parameter generated an assembly with the longest summed length of contigs and maximum total number of contigs, but it also produced the smallest N50 and mean contig length. Cap3 generated a relatively larger assembly size than Newbler (optimal parameter), but with shorter N50, mean contig length, and number of contig no less than 1,000 bp.

Another optimality criterion for a novel *de novo* assembled transcriptome we used in this study is how well the assembly represents protein sequences from soybean, the most related organism to *A. mongolicus* with sequenced genome (Table 2). A better assembler will return contigs that hit soybean data well, and will show a high coverage of the soybean protein datasets. The assembly generated by MIRA had the largest quantity of contigs with significant hits and soybean protein hits, while the assembly generated by Newbler (optimized parameter) had the largest quantity of contigs with 80% or greater coverage and soybean proteins with 80% or greater coverage.

Of the four assemblies we generated using the three assemblers, the assembly generated by Newbler (optimized parameter) was selected for further analysis, since it used the largest quantity of sequencing reads for assembly and had relatively large assembly size, longer contig length, and better assembly integrity and completeness. Another reason that we choose Newbler was due to its frequent use in *de novo* assembly of 454 pyrosequencing transcriptome projects [30].

### Characteristics of the *Ammopiptanthus* root transcriptome

**Figure 3:**
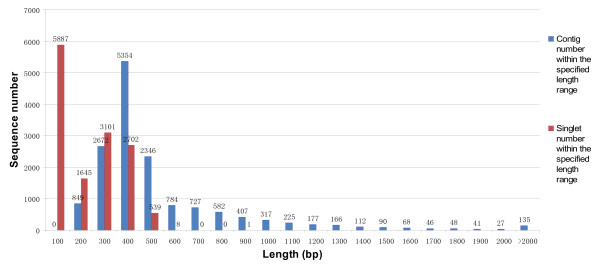
Size distribution of the contigs and singlets.

Using Roche Newbler (version 2.5.3) with optimized parameter, the 654,834 preprocessed sequencing reads were assembled into 29,056 unique sequences including 15,173 contig and 13,883 singlets. The sequencing coverage ranged from 2- to 17,162-fold, with an average 43.2-fold coverage. In total, 640,951 reads were assembled into contigs, accounting for 97.88% of the assembled reads and 95.38% of all sequencing reads. The contigs ranged from 100 to 4,659 bp, with an average size of 484 ± 349 bp. About 78.07% of the contigs were assembled from three or more reads. The size distribution for these contigs and singlets is shown in Figure 3.

**Figure 4:**
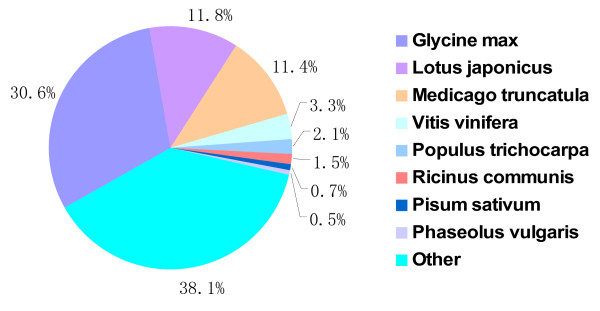
Species distribution of the top BLAST hits.

To study the sequence conservation of *A. mongolicus* in other plant species, we used BLAST [31] to align both contigs and singlets to the non-redundant database (nr) of the NCBI (the last update time: Jan. 23, 2011) using an E value threshold of 1e-10. Of 29,056 unique sequences, 8,790 (30.25%) had BLAST hits in nucleotide sequence database in NCBI. The majority of the annotated sequences corresponded to known nucleotide sequences of plant species, with 25.1%, 11.0%, 9.0%, 2.5%, and 1.9% matching with *Glycine maxLotus japonicusMedicago truncatulaVitis vinifera*, and *Populus trichocarpa* sequences, respectively (Figure 4).

### Frequency and distribution of EST-SSRs in the *A. mongolicus* root transcriptome

After screening EST-SSRs using MISA software in the 29,056 unique sequences (15,173 contigs, 13,883 singlets, and 9,958,274 bp total length), 1,827 SSRs distributed in 1,684 sequences were identified. The EST-SSR frequency in the *A. mongolicus* transcriptome was 5.80%, and the distribution density was 5.45 per kb. Two hundred and forty-six sequences contained more than two EST-SSRs. Based on the repeat motifs, all SSR loci were divided into mono-nucleotide, di-nucleotide, tri-nucleotide, tetra-nucleotide, penta-nucleotide, hexa-nucleotide, and multi-nucleotide. The most abundant type of repeat motif was tri-nucleotide (554, 30.32%), followed by mono-nucleotide (526, 28.80%), di-nucleotide (434, 23.75%), multi-nucleotide (198, 10.84%), tetra-nucleotide (75, 4.11%), hexa-nucleotide (19, 1.04%), and penta-nucleotide (21, 1.15%) repeat units (Table 3).

**Table 3:** **Frequency of EST-SSRs in*A. mongolicus***

Motif length	Repeat numbers							Total (%)		Total (%)	
	5	6	7	8	9	10	>10				
Mono-	-	-	-	-	-	-	526	526	28.08
Di-	-	146	73	53	39	26	97	434	23.75
Tri-	266	135	58	31	19	10	35	554	30.32
Tetra-	33	15	3	5	3	2	14	75	4.11
Penta-	9	4	3	-	-	-	3	19	1.04
Hexa-	13	3	1	1	1	1	1	21	1.15
Multi-	-	-	-	-	-	-	-	198	10.84
Total	321	303	138	90	62	39	150	1827	

The frequencies of EST-SSRs with different numbers of tandem repeats were calculated and are shown in Table 4. The SSRs with six tandem repeats (28.44%) were the most common, followed by five tandem repeats (26.92%), > 10 tandem repeats (13.36%), seven tandem repeats (13.26%), eight tandem repeats (8.50%), nine tandem repeats (5.87%), and ten tandem repeats (3.64%). The dominant repeat motif in EST-SSRs was AG/CT (26.72%), followed by AAG/CTT (13.77%), AAC/GTT (9.62%), AC/GT (9.31%), and AAT/ATT (8.81%) (Table 4). However, very few CG/CG (0.1%) repeats were identified in the databases.

**Table 4:** **Frequency of di- and tri-nucleotide EST-SSR repeat motifs in*A. mongolicus***

Repeat motif	Repeat numbers							Total	(%)
	5	6	7	8	9	10	>10		
AC/GT	-	38	16	9	5	7	17	92	9.31
AG/CT	-	73	42	30	28	15	76	264	26.72
AT/AT	-	34	15	14	6	4	4	77	7.79
CG/CG	-	1	-	-	-	-	-	1	0.10
AAC/GTT	39	22	9	6	3	5	11	95	9.62
AAG/CTT	63	34	18	6	9	-	6	136	13.77
AAT/ATT	45	29	7	5	-	1	-	87	8.81
ACC/GGT	22	8	2	7	3	2	-	44	4.45
ACG/CTG	5	3	2	-	-	-	-	10	1.01
ACT/ATG	22	13	8	1	2	-	11	57	5.77
AGC/CGT	19	6	5	2	-	1	-	33	3.34
AGG/CCT	25	5	2	-	1	-	2	35	3.54
AGT/ATC	23	15	5	4	1	1	5	54	5.47
CCG/CGG	3	-	-	-	-	-	-	3	0.30
Total	266	281	131	84	58	36	132	988	
(%)	26.92	28.44	13.26	8.50	5.87	3.64	13.36		

### Functional annotation

To find potential genes involved in drought response in our assembly, we used BLASTx [31] to align both contigs and singlets to the PlantGDB (http://www.plantgdb.org/), the protein database of soybean (Gmax_109, http://www.phytozome.net/soybean), and TAIR10 protein database using an E threshold of 1e-3 and protein identity no less than 30%.

Of 15,173 contigs, 6,486 (42.75%) had the BLAST hits to known proteins in PlantGDB (Table 5).

**Table 5:** 454 EST matches to annotated protein databases

Database	Contig (15,173)	Singlets (13,883)	Combined Set (29,056)
PlantGDB	6,486 (43%)	3,285 (24%)	9,771 (34%)
Gmax_109	5,146 (34%)	2,327 (17%)	7,473 (26%)
TAIR10	4,487 (30%)	2,200 (16%)	6,687 (23%)

Numbers and percentages of 454 ESTs in the assembled contigs, singlets, and the combined sequence set with matches to known proteins in BLASTx searches of three annotated protein databases (PlantGDB, Gmax_109, and TAIR10)

As expected, a remarkably lower percentage of the shorter singlet reads had BLAST hits to PlantGDB proteins. Of 13,883 singlet reads, 3285 (23.66%) had blast hits to PlantGDB proteins (Table 5). Smaller percentages of contigs and singlets had BLAST hits to the Gmax_109 and TAIR10 database (Table 5). This seemingly low percentage of BLAST hits is partially due to the shortage of protein sequences from Leguminosae woody plants in the public database, although annotation of only 30%-40% of sequences is common in analyses of large EST collections [32,33]. Nonetheless, BLAST searches identified a total of approximately 9,771 unique protein accessions, indicating that our transcriptome assembly datasets represented a substantial fraction of *A. mongolicus* root genes.

**Figure 5:**
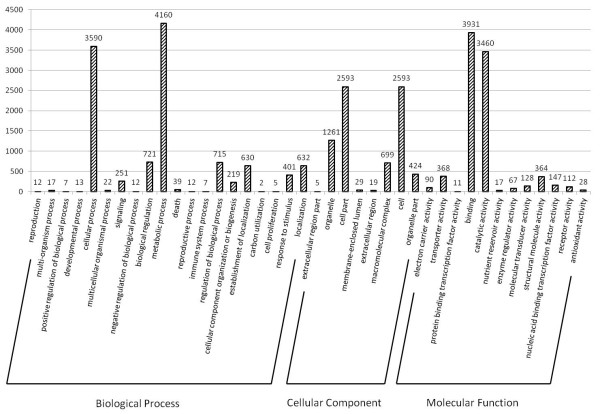
**Histogram presentation of Gene Ontology classification.** The results are summarized in three main categories: biological process, cellular component, and molecular function. The right y-axis indicates the number of genes in a category. The left y-axis indicates number of unique sequences in a specific category.

Gene ontology assignments were used to classify the functions of the *A. mongolicus* transcripts. Based on sequence homology, the 9,771 annotated sequences, which had BLAST hits to PlantGDB proteins, were categorized into 40 functional groups (Figure 5). In each of the three main categories (biological process, cellular component, and molecular function) of the GO classification, “metabolic process”, “cell & cell part”, and “binding” terms were dominant, respectively. We also noticed a high-percentage of genes from categories of “cellular process”, “organelle”, and “catalytic activity” and only a few genes from terms of “carbon utilization”, “cell killing”, “extracellular region part”, and “protein binding transcription factor activity” (Figure 5).

**Figure 6:**
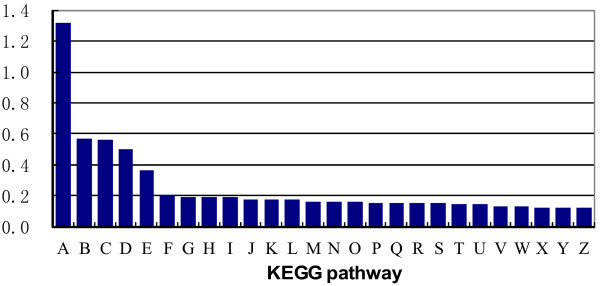
**Histogram presentation of KEGG classification of 9,771 annotated sequences.** A-Z are the top 26 KEGG pathways. The y-axis indicates the percentage of unique sequences assigned to a specific pathway in all unique sequences. The x-axis indicates the KEGG pathway. A, metabolic pathways; B, ribosome; C, biosynthesis of secondary metabolites; D, microbial metabolism in diverse environments; E, ubiquitin mediated proteolysis; F, glycine, serine, and threonine metabolism; G, protein processing in endoplasmic reticulum; H, spliceosome; I, RNA transport; J, glycerolipid metabolism; K, aminoacyl-tRNA biosynthesis; L, citrate cycle (TCA cycle); M, glycerophospholipid metabolism; N, cell cycle - yeast; O, phagosome; P, plant-pathogen interaction; Q, lysosome; R, cell cycle; S, endocytosis; T, glycolysis/gluconeogenesis; U, starch and sucrose metabolism; V, mRNA surveillance pathway; W, cysteine and methionine metabolism; X, oxidative phosphorylation; Y, RNA degradation; Z, synaptic vesicle cycle.

To identify the biological pathways that are active in root of *A. mongolicus*, we mapped the 9,771 annotated sequences (annotation by PlantGDB) to the reference canonical pathways in Kyoto Encyclopedia of Genes and Genomes (KEGG) [34] and the top 26 KEGG pathways are shown in Figure 6. The pathways with most representation by the unique sequences were “metabolic pathways”, “Ribosome”, and “Biosynthesis of secondary metabolites” (Figure 6). These results indicate that the diversifying metabolic processes are active in *A. mongolicus* root, and a variety of metabolites are synthesized in the root. In short, these annotations provide a valuable resource for investigating specific processes, functions, and pathways and facilitate the identification of novel genes involved in drought stress tolerance in root of *A. mongolicus*.

### Expression analysis of genes possibly involved in drought response in *A. mongolicus* root

**Figure 7:**
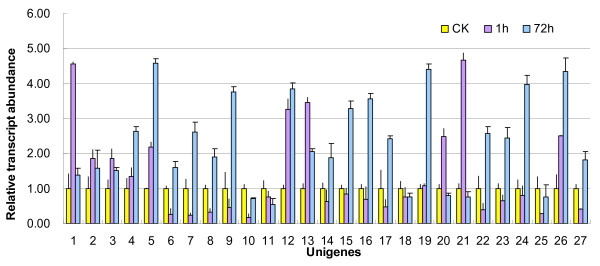
**Expression profiles of 27 drought-responsive unigenes.** Quantitative real-time PCRs were performed to analyze the expression profiles of 27 unigenes from four GO category under 1 h and 72 h exposure to 20% PEG-6000 treatment. 1, sdq_isotig00642; 2, sdq_isotig01704; 3, sdq_isotig11437; 4, sdq_isotig01576; 5, sdq_isotig02883; 6, sdq_isotig00259; 7, sdq_isotig01086; 8, sdq_isotig07386; 9, sdq_isotig11592; 10, sdq_isotig01905; 11, sdq_isotig10416; 12, sdq_isotig08490; 13, sdq_isotig01610; 14, sdq_isotig00634;15, sdq_isotig11067; 16, sdq_isotig07261;17, sdq_isotig06338; 18, sdq_isotig00577; 19, sdq_isotig04813; 20, sdq_isotig02931;21, sdq_isotig00833; 22, sdq_isotig01131; 23, sdq_isotig01737;24, sdq_isotig03894; 25, sdq_isotig07698; 26, sdq_isotig0699; 27, sdq_isotig00917.

To identify drought responsive genes, 27 unigenes were selected from the unique sequences classified in GO categories “response to osmotic stress” (unigene 1–11 in Figure 7), “response to oxidative stress” (unigene 12–18 in Figure 7), “response to hormone stimulus” (unigene 19–21 in Figure 7), and “response to light stimulus” (unigene 23–27 in Figure 7). Quantitative real-time PCR assay were performed using the primers ( Additional files 1) designed according to these unigenes to monitor their expression profiles under 1 h and 72 h exposure to 20% PEG-6000 treatment.

The results indicated the expression of 27 unigenes that showed significantly up-regulated or down-regulated patterns at least at one time-point under exposure to PEG-6000 treatment. According to their expression patterns, the 27 drought-responsive unigenes were classified into four groups ( Additional file 2), *U-I* increased at both 1 h and 72 h, *U-II* increased at 1 h but decreased at 72 h, *-I* decreased at both 1 h and 72 h, and *D-II* decreased at 1 h but increased at 72 h. Among the 27 unigenes responsive to PEG-6000 treatment, 12 showed *D-II* pattern and 9 shows *U-I* pattern; in contrast, four unigenes behaved *D-I* pattern and only two unigenes behaved *U-II* pattern.

## Discussion

As a relic survivor of the evergreen broadleaf forest of central Asia from the Tertiary period, *A. mongolicus* can tolerate serious drought stress. The stress tolerance of *A. mongolicus* may not only associated with the epicuticular wax and stomata, which reduce the water evaporation, but also the deep flourishing root system, which enables the pant to absorb water deep below the soil surface. Our previous work (unpublished observations) revealed that, comparing with the shoot, the physiological index (*i.e.*, proline content and antioxidants) in the root of *A. mongolicus* responded to the drought stress faster and more significant. Investigation of the gene expression regulation network under drought stress will be helpful to understand the biochemical and physiological adaptation process in *A. mongolicus*, since there are only 748 *Ammopiptanthus* ESTs in GenBank. In the present study, large-scale root-specific transcriptome data were obtained by high throughput 454 sequencing as the first step of our endeavor to provide a clear insight into the molecular mechanism of drought tolerance in *A. mongolicus*.

Most plant transcriptomic studies sequenced the pooled cDNA samples from different tissues [33,35-37], or assembly transcriptomic data using sequencing reads from different tissues [38], only a few work perform root-specific transcriptomic sequencing and assembly [39,40]. Although more extensive transcriptomic data can be obtained using the former strategy, more accurate data can be produced using the later method, since alternative splicing may exist in different tissues [41], which will make the contig assembly difficult. Furthermore, the tissue-specific transcriptomic study will provided a good reference data for gene expression profiling, especially in non-model plant.

There are three high throughput sequencing methods that can be used for transcriptomic study, including the classic and the most popular 454 pyrosequencing, and the low-cost solexa sequencing, which were employed more and more frequently in recent years [30]. In this study 454 pyrosequencing was adopted to gain a longer and more reliable transcriptomic dataset.

Choosing suitable assembler and parameters is critical to getting a better assembly performance, which is even more important in transcriptomic studies in non-model organisms. However, most previous analyses of transcriptomic data generated by Roche 454 pyrosequencing have almost always used only one software program for assembly [30] except a recent study [38] in which the assembles from six assemblers were compared including Velvet, ABySS, MIRA, Newbler v2.3, Newbler v2.5p, CLC, and TGICL. In the present study, we compared the assembly from the three most frequently used assemblers, *i.e.* MIRA, Newbler v2.5.3, and Cap3 [30], since Velvet and ABySS are not developed for relatively long sequence assembly.

Evaluation of assembly performance is a challenging work, especially in non-model organisms. We adopted two groups of index for assembly evaluation according to an earlier study [38]. The first group of index included total number of reads used in the assembly, number of contigs generated, N50 length of contigs, number of contigs, mean contig length, and summed contig length (Table 2). The second group of index was obtained by comparing with the soybean protein datasets (Table 2).

Indeed, the comparison (Table 2) revealed that the assemblies generated from different software programs showed advantages and disadvantages in different aspect. Anyway, the assembly generated by Newbler (optimized parameter) was selected for further analysis according to the comparison result and its frequent application.

From 672,002 sequence reads, 29,056 unigenes were assembled, which consisted of 15,173 contigs and 13,883 singlets from drought-stressed and unstressed roots of *A. mongolicus*. Although a high number of unigenes were not long enough to cover the complete protein-coding regions as revealed by BLASTX aligment, up to now, the dataset we reported here still provided the largest dataset of different genes representing a substantial part of the transcriptome of *A. mongolicus*, which probably embraces the majority part of genes involved in the sophisticated regulation networks for sensing and acclimating the water-deficit soil environment.

Relatively large portion (97.26%) of reads were assembled into contigs, which is significantly higher than that reported for several other recent 454 transcriptome assemblies (*e.g.*, 48% [33]; 88% [16]; and 90% [32]). As a consequence, our *A. mongolicus* root transcriptomic data showed a relatively high coverage depth (ranging from 1 to 17,162-fold with an average 45.3-fold), comparing with some other transcriptomic data from other plants (*e.g.*, 3.6 [33]; 8 [39]; 3.1 [42]). This may indicate that half-plate 454 pyrosequencing is deep enough for root transcriptome. Nonetheless, our contig length (484 bp) is not higher than other transcriptomic data (*e.g.*, 345 [43]; 364 [20]; 452 [33]; 526 [39]; and 618 [37]).

SSRs consist of tandem repeats of short (1–6 bp) nucleotide motifs [44]. These repeat sequences are distributed throughout the genome. Polymorphism revealed by SSRs results from variation in repeat number, which primarily results from slipped-strand mispairing during DNA replication. Thus, SSRs reveal much higher levels of polymorphism than most other marker systems [45,46]. SSRs have proven to be more reliable than other markers, and the utility of SSRs in genetics studies is well established.

We screened 1,827 SSR loci, and EST-SSR frequency in the *A. mongolicus* transcriptome was 5.80%. The AG/CT and AAG/CTT repeat motifs were the most SSR motifs in all nucleotide repeat motifs, and tri-nucleotide repeats was the most frequent type of SSR motif. This finding is consistent with the results reported in cereals such as rice (*Oryza sativa*), wheat (*Triticum aestivum*), and barley (*Hordeum vulgare*) [47]. Di-nucleotide repeats were the most abundant class of SSRs in many plant species such as *Arabidopsis*, peanut (*Arachis hypogaea*), canola (*Brassica napus*), sugar beet (*Beta vulgaris*), cabbage (*Brassica oleracea*), soybean (*Glycine max*), sunflower (*Helianthus annuus*), sweet potato (*Ipomoea batatas*), pea (*Pisum sativum*), and grape (*Vitis vinifera*) [24,48]. Among the di-nucleotide repeats, AG/CT was the most frequent motif in our study, whereas CG/CG motif was very rare. Among the tri-nucleotide repeats, the AAG/CTT motif was the most frequent one. Our results are consistent with those in other plant species [24,48-50]. In plants, CT and CTT repeats are found in both transcribed regions and 5'-untranslated regions (UTRs); CT microsatellites in 5' UTRs may be involved in antisense transcription and play a role in gene regulation [51].

Drought tolerance is a complex trait and involves multiple mechanisms that act in combination to avoid or tolerate periods of water deficit. It is well-established that, under drought stress, the genes involved in osmotic and redox homeostasis will be regulated and hormones such as ABA will participate in the readjustment process. Recently, light-mediated root growth is believed to be relevant to drought tolerance of root [52]. Hence, 27 unigenes classified in GO categories “response to osmotic stress”, “response to oxidative stress”, “response to hormone stimulus”, and “response to light stimulus” were selected for further expression analysis. As expected, some ion channel and transporter genes (*i.e.*, sdq_isotig00642, sdq_isotig01704, sdq_isotig11437, sdq_isotig00259, sdq_isotig01086, and sdq_isotig10416), as well as several anti-oxidant (*i.e.*, sdq_isotig08490, sdq_isotig01610, sdq_isotig00634, sdq_isotig11067, sdq_isotig07261, and sdq_isotig00577) were shown to be involved into the drought response. Quantitative real-time PCR also revealed that the gene expressions of some blue light photoreceptor NPH3 (*i.e.*, sdq_isotig01737, sdq_isotig01131, sdq_isotig3894, and sdq_isotig07698) and an interacting protein of NPH1 (sdq_isotig00917) were regulated under drought stress, which confirmed the relevance of light-mediated root growth to drought tolerance of root. Furthermore, an ethylene receptor gene was shown to be up-regulated only at 72 h, and an auxin receptor and an auxin induced gene, IAA9, were up-regulated only at 1 h, suggesting that the ethylene and auxin may participate in drought response of root in *A. mongolicus*.

Our study identified 27 drought responsive genes. The functions of these genes in drought tolerance of root will be analyzed by transgenic study. At the same time, more drought response genes will be discovered by digital gene expression analysis based on the transcriptome data obtained in this study. We are confident that more light will soon be shed on the adaptive significance of *A. mongolicus* root for plant adaptation to the drought environment.

## Conclusions

*Ammopiptanthus mongolicus* is an ecologically important plant species in Mid-Asia desert and exhibits substantial tolerance to drought condition. Insufficient transcriptomic and genomic data in public databases has limited our understanding of the molecular mechanism underlying the stress tolerance of *A. mongolicus*. The 29,056 unique sequences in this 454 EST collection represent a major transcriptomic level resource for *A. mongolicus*, and will be useful for further functional genomics study in *Ammopiptanthus* genus. The thousands of SSR markers predicted in our 454 ESTs should facilitate population genomic studies in *Ammopiptanthus*. The potential drought stress related transcripts identified in this study provide a good start for further investigation into the drought adaptation in *Ammopiptanthus*. Additionally, our results also highlight the utility of high-throughput transcriptome sequencing as a fast and cost-effective approach for marker development and gene discovery in non-model species.

## Methods

### Sample preparation and 454 pyrosequencing

Seeds of *A. mongolicus* (collected from the desert region in Zhongwei County, Ningxia Autonomous Region, China) were soaked in water for 48 h at 25 °C and then sown in 9 cm diameter commercial pots containing vermiculite and perlite (with 1:1 ratio of vermiculite to perlite) in a greenhouse at approximately 25 °C and 35% relative humidity under a photosynthetic photon flux density of 120 μmol m^-2^ s^-1^ with a photoperiod of 16 h light and 8 h dark. The plantlets were watered in a three-day interval with half strength of Hoagland’s solution. Two weeks after germination, the seedlings were divided into two groups. The first group served as the control (CK) whilst the second (T) was irrigated with 20% PEG-6000. The roots of both samples were harvested after 72 h and used for RNA extraction immediately.

Total RNA extraction, mRNA purification, and cDNA library construction were conducted by LC Sciences (Houston, TX, USA). In brief, total RNA was obtained from roots using the total RNA purification kit (LC Sciences, Houston, TX, USA) as instructed, treated with RNAase free DNAase, and re-purified with the RNeasy kit (Qiagen, Valencia, CA, USA) following the manufacturer’s protocol. Equal quantity of RNA from both CK and T samples were blended for cDNA library construction.

cDNA synthesis was performed using SMART II™ cDNA Synthesis kit (Clontech Laboratories, Inc., Mountain View, CA, USA) following manufacturer’s recommendations. Double stranded cDNA was separated on a 2% agarose gel, and the cDNA with a length no less than 100 bp was separated by gel extraction. The concentration of cDNA was determined using Bioanalyzer 2100 (Agilent Technologies, Inc., Waldbronn, Germany). Approximately 5 μg of cDNA sample was sheared *via* sonication into small fragments, and then Roche GS-FLX 454 pyrosequencing was conducted according to the manufacturer’s recommendations.

### *De novo* assembly

Raw data generated from 454 pyrosequencing were preprocessed to remove nonsense sequences including (1) adapters that were added for reverse transcription and 454 sequencing, (2) primers, (3) very short (<50 bp) sequences, and (4) low quality sequences using Lucy, Seqclean and Newbler program.

The preprocessed sequences were then assembled using assembly program with default or optimal parameter. Among various programs available, we used publicly available programs Cap3 (http://seq.cs.iastate.edu/cap3.html), and MIRA (version 3.2.1; http://sourceforge.net/projects/miraassembler/), as well as GS *De novo* Assembler (Newbler v2.5.3; http://www.454.com/products-solutions/analysis-tools/gs-de-novoassembler.asp) supplied with the GS FLX Titanium sequencer.

To examine the coverage of the sequences, all unique sequences (contigs and singlets) generated from different assembler with default or optimal parameter were compared with the publicly available soybean protein dataset (Phytozome v7.0, http://www.phytozome.net/) using Blastx program and a typical cutoff value of E < 1e-5 was used.

### Functional annotation and EST-SSRs marker identification

Sequence assembly and annotation were carried out by Zhongxin Biotechology Shanghai Co., Ltd (Shanghai, China). For annotation, all unique sequences were searched against protein database PlantGDB (http://www.plantgdb.org/, the update time: April 20, 2011), soybean (Gmax_109, http://www.phytozome.net/soybean, version: 7.0, the last update time: Mar. 31, 2011) [53], and TAIR10 protein database [54] using a threshold of E < 1e-5 and protein identity >30%. Gene ontology analysis was conducted on the annotated sequences through custom Perl script.

Pathway assignments were carried out according to KEGG mapping [55] using custom Perl script. MISA (http://pgrc.ipk-gatersleben.de/misa/) was used to identify the potent EST-SSR markers in all unique sequences. Dinucleotides repeats of more than six times and trinucleotide, tetranucleotide, pentanucleotide, and hexanucleotide repeats of more than five times were considered as the search criteria for SSRs in MISA script.

### Quantitative real-time PCR analysis

Approximately 1 μg of DNase I-treated total RNA was converted into single-stranded cDNA using M-MLV Reverse Transcriptase (Promega, USA). The cDNA products were then diluted 50-fold with deionized water before using as a template in real-time PCR. The quantitative reaction was performed on an MyiQ2 two-color real-time PCR detection system (Bio-Rad Laboratories, Hercules, CA, USA) using the SsoFast EvaGreen Supermix (Bio-Rad Laboratories, Hercules, CA, USA). The reaction mixture (20 μL) contained 2× SsoFast EvaGreen Supermix, 0.9 μM each of the forward and reverse primers, and 1 μL of template cDNA. PCR amplification was performed under the following conditions: 95 °C for 30 s, followed by 40 cycles of 95 °C for 5 s and 60 °C for 10 s. Two independent biological replicates for each sample and three technical replicates of each biological replicate were analyzed in quantitative real-time PCR analysis. The gene expressions of selected unigenes were normalized against an internal reference gene, 18SrRNA. The relative gene expression was calculated using the 2^-ΔΔCt^ method [56]. All primers used in this study are listed in Additional File 1.

## Competing interests

The authors declare that they have no competing interests.

## Authors’ contributions

The study was conceived by YZ, JF, and FG. JF collected the seeds of *A. mongolicus*. The plant material preparation and gene expression analyses were carried out by RL. YZ, FG, and HL contributed to data analysis, bioinformatics analysis, and manuscript preparation. All authors had read and approved the final manuscript.

## Supplementary Material

Additional file 1 **The primers used in quantitative real-time PCR analysis.** This table lists all the primers used in quantitative real-time PCR analysis. Click here for file

Additional file 2 **The annotation, GO category and relative transcript abundance of the 27 unigenes selected for quantitative real-time PCR analysis.** This table lists the annotation, GO category, relative transcript abundance of the 27 unigenes selected for quantitative real-time PCR analysis. Click here for file
