# Supplementary material for: De novo sequencing and analysis of root transcriptome using 454 pyrosequencing to discover putative genes associated with drought tolerance in Ammopiptanthus mongolicus
Source: BMC Genomics. 2012 Jun 21;13:266. doi: 10.1186/1471-2164-13-266 (PMC3407029; doi:10.1186/1471-2164-13-266)
Supplement: Additional file 1 — The primers used in quantitative real-time PCR analysis. This table lists all the primers used in quantitative real-time PCR analysis. [file 1471-2164-13-266-S1.doc]

Additional file 1 - The primers used in quantitative real-time PCR analysis

| Unigenes or internal reference gene | Primers (5' to 3') | Base number (nt) |
| --- | --- | --- |
| 18S rRNA F | CGGCTACCACATCCAAGGAA | 20 |
| 18S rRNA R | GCTGGAATTACCGCGGCT | 18 |
| sdq_isotig00642 F | CTCCCCAGAAATGGTGAAGA | 20 |
| sdq_isotig00642 R | CAGCTGTTGGTGCTGAGGTA | 20 |
| sdq_isotig01704 F | CCGCAGTTTTCTGAAAGAGG | 20 |
| sdq_isotig01704 R | CAGCCCAAGTTCTCCATTGT | 20 |
| sdq_isotig11437 F | GGCAGTGGAACCACAGTTCT | 20 |
| sdq_isotig11437 R | ACGCCTTCTGACTTGTGGAC | 20 |
| sdq_isotig01576 F | GCTGGATTTGCCACAAAAAT | 20 |
| sdq_isotig01576 R | GGAGCATACCCATTTGAGGA | 20 |
| sdq_isotig02883 F | TGATACAAAACGGCCAATGA | 20 |
| sdq_isotig02883 R | TTCTATGGCTGTCGATGTGC | 20 |
| sdq_isotig00259 F | GCCTTCGCAGTAGCTTCATC | 20 |
| sdq_isotig00259 R | CGAAGAGGACCGCTTAACTG | 20 |
| sdq_isotig00917 F | TGCAGCCAAGTTTTGCTATG | 20 |
| sdq_isotig00917R | ATCTGCGGACAATTTGAAGG | 20 |
| sdq_isotig01086 F | ATCCTTGCATAGAGGGCACA | 20 |
| sdq_isotig01086 R | AACTGCGACCCAAACAGTCT | 20 |
| sdq_isotig07386 F | CTGCACTCCCAGCCTAAATC | 20 |
| sdq_isotig07386 R | GTGGGTCCTCTGGATCTTCA | 20 |
| sdq_isotig11592 F | TGGCATACACTATCTCCCCACT | 22 |
| sdq_isotig11592 R | CCTGCAGACCTGATGAATGA | 20 |
| sdq_isotig1905 F | ATCTCAATGAGCGCCACTCT | 20 |
| sdq_isotig1905 R | TGCCAGGTCACAAGAAGACA | 20 |
| sdq_isotig10416 F | GTTTTGAGTACAGCGCACCA | 20 |
| sdq_isotig10416 R | GCCTTTCTTGTCAGCTCTGG | 20 |
| sdq_isotig08490 F | CCTTAGCCCAACTTCAACCA | 20 |
| sdq_isotig08490 R | TCCACCACACTTCGTGTAGC | 20 |
| sdq_isotig01610 F | TGTTGCCTCACAATGTGGTT | 20 |
| sdq_isotig01610 R | AAGTGGTGCTGCATTCTTCC | 20 |
| sdq_isotig00634 F | TTGGTGGCAAAATTTGGAGT | 20 |
| sdq_isotig00634 R | CACCACTTGGCCATCCTTAT | 20 |
| sdq_isotig11067 F | TTCTCAGCATCAGCCATCAC | 20 |
| sdq_isotig11067 R | GAGGCAGAGCAGAGAGCATT | 20 |
| sdq_isotig07261 F | TTGCAGAAAGCACTTGGAGA | 20 |
| sdq_isotig07261 R | CACAGCCCTCACACACAAAC | 20 |
| sdq_isotig06338 F | TACGCAGCCCAATTACAACA | 20 |
| sdq_isotig06338 R | TGGTAATGGCAGCAATGAAA | 20 |
| sdq_isotig00577 F | GTGCACATTCTCTTGCTGGA | 20 |
| sdq_isotig00577 R | GGGAATTGGAGGAGGTTTTC | 20 |
| sdq_isotig04813 F | ACAAATTGCAAGGCCAAGTC | 20 |
| sdq_isotig04813 R | ATTCCTTCCAGTGCCAAGTG | 20 |
| sdq_isotig02931 F | CTTTCGGTAACACGGCACTT | 20 |
| sdq_isotig02931 R | TGGTGCATCTTTCCTCTTCC | 20 |
| sdq_isotig00833 F | CAGTTCTTGCCTCCTCCAAG | 20 |
| sdq_isotig00833 R | GGCTGAGGATCATGAAGAGC | 20 |
| sdq_isotig01131 F | CACCAGTTTTCCCCTCTGAA | 20 |
| sdq_isotig01131 R | AACCGAGAACTGAAGCTGGA | 20 |
| sdq_isotig01737 F | GGGCACTTCAAACCTGTGAT | 20 |
| sdq_isotig01737 R | TCCACAAAACACTTCCACCA | 20 |
| sdq_isotig03894 F | TCACTTGACCTGCAAGCATC | 20 |
| sdq_isotig03894 R | TGCCCTGTAAAGTCCATCGT | 20 |
| sdq_isotig07698 F | ATGACCATTTGACCTGCACA | 20 |
| sdq_isotig07698 R | GGATTCCATTGCTACGCTTC | 20 |
| sdq_isotig0699 F | CTGCCCCACTTTTAGCAGAG | 20 |
| sdq_isotig0699 R | CAGAGTGGGGACTCGTTGTT | 20 |
