# Supplementary material for: De novo sequencing and analysis of root transcriptome using 454 pyrosequencing to discover putative genes associated with drought tolerance in Ammopiptanthus mongolicus
Source: BMC Genomics. 2012 Jun 21;13:266. doi: 10.1186/1471-2164-13-266 (PMC3407029; doi:10.1186/1471-2164-13-266)
Supplement: Additional file 2 — The annotation, GO category and relative transcript abundance of the 27 unigenes selected for quantitative real-time PCR analysis. This table lists the annotation, GO category, relative transcript abundance of the 27 unigenes selected for quantitative real-time PCR analysis. [file 1471-2164-13-266-S2.doc]

Additional file 2 - The annotation, GO category and relative transcript abundance of the 27 unigenes

| Number | Unigenes | Annotation (Tair10) | GO category | Fold change | | Classification of gene expression patterm |
| --- | --- | --- | --- | --- | --- | --- |
|  |  |  |  | 1h | 72h |  |
| 1 | sdq_isotig00642 | AT3G01280.1, voltage-dependent anion channel (VDAC) | response to osmotic stress | 4.56 | 1.39 | *U-I* |
| 2 | sdq_isotig01704 | AT2G23980.1, member of Cyclic nucleotide gated channel family | response to osmotic stress | 1.85 | 1.58 | *U-I* |
| 3 | sdq_isotig11437 | AT3G02050.1, potassium transporter KUP3p (KUP3) | response to osmotic stress | 1.85 | 1.52 | *U-I* |
| 4 | sdq_isotig01576 | AT1G10940.2, plant protein kinase similar to the calcium / calmodulin-dependent protein kinase subfamily and the SNF1 kinase subfa | response to osmotic stress | 1.34 | 2.63 | *U-I* |
| 5 | sdq_isotig02883 | AT1G09010.1, glycoside hydrolase family 2 protein; FUNCTIONS IN: cation binding, hydrolase activity, hydrolyzing O-glycosyl compounds, catalytic activity | response to osmotic stress | 2.19 | 4.58 | *U-I* |
| 6 | sdq_isotig00259 | ATCG00480.1, beta subunit of ATP synthase | response to osmotic stress | 0.25 | 1.6 | *D-II* |
| 7 | sdq_isotig01086 | ATCG00470.1, ATPase epsilon subunit | response to osmotic stress | 0.23 | 2.61 | *D-II* |
| 8 | sdq_isotig07386 | AT4G40010.1, encodes a member of SNF1-related protein kinases (SnRK2) whose activity is activated by ionic (salt) and non-ionic (mannitol). | response to osmotic stress | 0.32 | 1.89 | *D-II* |
| 9 | sdq_isotig11592 | AT5G66880.1, encodes a member of SNF1-related protein kinases (SnRK2) whose activity is activated by ionic (salt) and non-ionic (mannitol) osmotic stress | response to osmotic stress | 0.45 | 3.75 | *D-II* |
| 10 | sdq_isotig01905 | AT1G76180.2, Encodes a dehydrin protein whose expression is induced early on in response to dehydration stress | response to osmotic stress | 0.17 | 0.71 | *D-I* |
| 11 | sdq_isotig10416 | AT1G22150.1, sulfate transporter Sultr1;3 | response to osmotic stress | 0.75 | 0.55 | *D-I* |
| 12 | sdq_isotig08490 | AT2G41480.1, Peroxidase superfamily protein; FUNCTIONS IN: peroxidase activity, heme binding | response to oxidative stress | 3.25 | 3.85 | *U-I* |
| 13 | sdq_isotig01610 | AT2G43350.2, ATGPX3 | response to oxidative stress | 3.46 | 2.04 | *U-I* |
| 14 | sdq_isotig00634 | AT1G63460.1, ATGPX8 | response to oxidative stress | 0.63 | 1.87 | *D-II* |
| 15 | sdq_isotig11067 | AT5G06430.1, Thioredoxin superfamily protein | response to oxidative stress | 0.85 | 3.27 | *D-II* |
| 16 | sdq_isotig07261 | AT5G13810.1, Glutaredoxin family protein | response to oxidative stress | 0.7 | 3.57 | *D-II* |
| 17 | sdq_isotig06338 | AT5G67400.1, root hair specific 19 (RHS19); FUNCTIONS IN: peroxidase activity, heme binding | response to oxidative stress | 0.48 | 2.42 | *D-II* |
| 18 | sdq_isotig00577 | AT4G04950.1, glutaredoxin 4 (GRX4) | response to oxidative stress | 0.75 | 0.76 | *D-I* |
| 19 | sdq_isotig04813 | AT1G66340.1, Ethylene receptor. Has histidine kinase activity | response to hormone stimulus | 1.08 | 4.41 | *U-I* |
| 20 | sdq_isotig02931 | AT1G12820.1, Auxin receptor involved in primary and lateral root growth inhibition in response to nitrate. Target of miR393 | response to hormone stimulus | 2.49 | 0.79 | *U-II* |
| 21 | sdq_isotig00833 | AT5G65670.2, auxin (indole-3-acetic acid) induced gene, IAA9 | response to hormone stimulus | 4.66 | 0.75 | *U-II* |
| 22 | sdq_isotig01131 | AT3G08570.1, Phototropic-responsive NPH3 family protein | response to light stimulus | 0.38 | 2.56 | *D-II* |
| 23 | sdq_isotig01737 | AT1G30440.1, Phototropic-responsive NPH3 family protein | response to light stimulus | 0.64 | 2.43 | *D-II* |
| 24 | sdq_isotig3894 | AT3G44820.1, Phototropic-responsive NPH3 family protein | response to light stimulus | 0.8 | 3.98 | *D-II* |
| 25 | sdq_isotig07698 | AT5G47800.1, Phototropic-responsive NPH3 family protein | response to light stimulus | 0.27 | 0.75 | *D-I* |
| 26 | sdq_isotig0699 | AT3G08570.1, Phototropic-responsive NPH3 family protein | response to light stimulus | 2.51 | 4.33 | *U-I* |
| 27 | sdq_isotig00917 | AT5G64330.1, blue light response signaling pathway; interacts with the blue light photoreceptor NPH1 | response to light stimulus | 0.4 | 1.82 | *D-II I* |
